# 'A major lobbying effort to change and unify the excise structure in six Central American countries': How British American Tobacco influenced tax and tariff rates in the Central American Common Market

**DOI:** 10.1186/1744-8603-7-15

**Published:** 2011-05-19

**Authors:** Chris Holden, Kelley Lee

**Affiliations:** 1 Department of Social Policy and Social Work, University of York, Heslington, York, YO10 5DD, UK; 2 Department of Global Health and Development, London School of Hygiene and Tropical Medicine, 15-17 Tavistock Place, London, WC1H 9SH, UK

## Abstract

**Background:**

Transnational tobacco companies (TTCs) may respond to processes of regional trade integration both by acting politically to influence policy and by reorganising their own operations. The Central American Common Market (CACM) was reinvigorated in the 1990s, reflecting processes of regional trade liberalisation in Latin America and globally. This study aimed to ascertain how British American Tobacco (BAT), which dominated the markets of the CACM, sought to influence policy towards it by member country governments and how the CACM process impacted upon BAT's operations.

**Methods:**

The study analysed internal tobacco industry documents released as a result of litigation in the US and available from the online Legacy Tobacco Documents Library at http://legacy.library.ucsf.edu/. Documents were retrieved by searching the BAT collection using key terms in an iterative process. Analysis was based on an interpretive approach involving a process of attempting to understand the meanings of individual documents and relating these to other documents in the set, identifying the central themes of documents and clusters of documents, contextualising the documentary data, and choosing representative material in order to present findings.

**Results:**

Utilising its multinational character, BAT was able to act in a coordinated way across the member countries of the CACM to influence tariffs and taxes to its advantage. Documents demonstrate a high degree of access to governments and officials. The company conducted a coordinated, and largely successful, attempt to keep external tariff rates for cigarettes high and to reduce external tariffs for key inputs, whilst also influencing the harmonisation of excise taxes between countries. Protected by these high external tariffs, it reorganised its own operations to take advantage of regional economies of scale. In direct contradiction to arguments presented to CACM governments that affording the tobacco industry protection via high cigarette tariffs would safeguard employment, the company's regional reorganisation involved the loss of hundreds of jobs.

**Conclusions:**

Regional integration organisations and their member states should be aware of the capacity of TTCs to act in a coordinated transnational manner to influence policy in their own interests, and coordinate their own public health and tax policies in a similarly effective way.

## Background

Within the growing literature on trade and health, limited attention to date has been given to regional trade integration. Analysis of trade and tobacco has tended to focus on the effects of liberalisation in increasing competition and thus consumption [1]. Yet processes of regional trade integration may entail changes to tariff and tax rates and structures that are more complex than simply generalised reductions. Furthermore, such regional processes hold significant implications for the ways in which transnational corporations organise their manufacturing and other operations, which may also impact upon public health. Such processes have not been widely studied, yet are integral to the transnational reorganisation of the tobacco industry and its capacity to continue to thrive in a rapidly changing world economy. For example, the limited analysis to date has demonstrated that transnational tobacco companies (TTCs) have responded to processes of regional trade integration both by attempting to influence public policy and by restructuring their own operations. In the Andean Pact during the 1990s, British American Tobacco (BAT) reorganised its operations so as to act more strategically at the regional level, whilst also attempting to influence policy [2].

BAT's behaviour in the Andean Pact is consistent with studies which have described systematic attempts by TTCs to influence public policies in a range of other settings, including in the Former Soviet Union [3,4], East Asia [5,6], and the United States [7]. In the US, TTCs have sought to influence legislators via the use of contract lobbyists; substantial contributions to legislators' campaigns, political caucuses and parties; the provision of gifts, honoraria, corporate hospitality and charitable donations; alliance with other interest groups; and the use of front groups [7,8]. TTCs have also gained access to the executive level of government in both developed and developing countries. BAT, for example, had a British Chancellor of the Exchequer as a non-executive director [8] and had 'close relationships with successive Kenyan presidents' [9]. Other routes to policy influence have included privileged access to trade officials, the employment of former civil servants, and the use of organised business groups such as the Multinational Chairmen's Group in the UK and the European Round Table [6]. Such studies demonstrate the routine use of lobbying and other mechanisms to influence policy by TTCs, often (though not always) with considerable success.

This article seeks to strengthen understanding of trade and tobacco at the regional level by analysing the activities of BAT in the Central American Common Market (CACM). As a means of promoting lasting peace in the region, following long periods of civil war in a number of member countries, the five countries of the CACM (Guatemala, El Salvador, Honduras, Nicaragua and Costa Rica), together with Panama, undertook to revitalise the organisation in the early 1990s [10]. This marked a shift, from an earlier 'closed regionalism' of the CACM based on import substitution industrialisation (ISI), to an 'open regionalism' based on liberal economic principles [11]. The latter also reflected a broader process of regional and sub-regional trade integration taking place in Latin America at that time [12]. In December 1990, the presidents of the five member countries committed themselves to the creation of an effective common market, with the opening of negotiations on a comprehensive regional customs and tariffs policy by March 1991 [10]. In October 1993, the five countries and Panama signed a protocol to the original CACM treaty of 1960 committing themselves to full economic integration, with a common external tariff of 20% for finished products, and 5% for raw materials and capital goods [10].

BAT dominated the Central American cigarette market during this period, with Philip Morris (PM) as its only regional rival. BAT subsidiaries had manufacturing monopolies in both Honduras and Nicaragua, and enjoyed the largest market share in Costa Rica, El Salvador and Panama (see Table 1) [13]. Only in Guatemala did PM have a marginally larger market share than BAT. In this context, the process of regional trade integration was likely to have a particularly important impact upon BAT.

**Table 1:** BAT and PM market share in Central American countries, 1992, percent [Source: [13]]

	Guatemala	El Salvador	Honduras	Nicaragua	Costa Rica	Panama
BAT	43.7	72.3	100	100	68.3	67.7

PM	56.3	27.7			30.9	32.3

This article therefore has two interlinked aims: first, to ascertain how BAT attempted to influence public policy in relation to the process of regional trade integration in the CACM; and, second, to ascertain how that process led BAT to adapt its own operations. The article draws conclusions regarding the need for closer attention to regional trade integration in tobacco control.

## Methods

This study analysed internal tobacco industry documents available from the Legacy Tobacco Documents Library at http://legacy.library.ucsf.edu/. The release of these documents in 1998 allowed researchers unprecedented access to the inner workings of TTCs, their strategies and attempts to influence public policy [14]. The provenance and limitations of the documents have been discussed elsewhere [15-19]. Limitations include the fact that the legal process did not result in a comprehensive set of all company documents being made available [15]. For example, BAT documents do not include those of its subsidiary companies outside of the UK and the US. However, a vast range of correspondence between BAT headquarters and its subsidiaries is available, sufficient to allow an extensive analysis of corporate strategy in a large number of areas.

Documents were initially retrieved by searching BAT documents using the key search terms 'Central American Common Market' and 'CACM'. Resultant documents were then used to generate new search terms in an iterative process. Analysis was based on an approach to company document analysis adapted from Forster [20] and complemented by strategies for interpreting archival material recommended by Hill [21]. This involved attempting to understand the meanings of individual documents and relating these to other documents in the set, identifying the central themes of documents and clusters of documents, contextualising the documentary data, and choosing representative material in order to present findings. Reliability of interpretation was increased by both authors participating in the interpretative process, agreeing on central themes and selecting appropriate material to quote. Results are presented thematically and, within each central theme, chronologically.

Analysis of the documents revealed that BAT's attempts to influence public policy in relation to the regional integration process related overwhelmingly to tax and tariff rates. The analysis also confirmed that the integration process had a significant impact on BAT's structure within the region, particularly in relation to manufacturing operations. Results related to these key themes of tariff rates, excise taxes, and the reorganisation of operations are reported below.

## Results

### 'Ready access and close contact with government officials': Influencing tariff rates

In October 1991, a report was circulated within BAT on the CACM which outlined developments in the negotiations among member countries to create a common market [22]. The document discussed the goal of creating a customs union, with a common external tariff and the abolition of tariffs between member countries, although it noted that at that time no specific proposals had been made to remove tariffs between member states. The report's analysis of member countries' current tariff rates on cigarettes imported from outside the CACM concluded that the proposed common external tariff of 20% would 'effectively halve the amount of protection given to domestic cigarette manufacture' [22].

For BAT, the proposed external tariff rate was a problem as the Central American market would then 'become more attractive to overseas manufacturers' [22]. One strategy identified to protect the company's market share was to move from its then current structure, whereby cigarettes were manufactured separately in each country by national operating companies, to a regional structure whereby manufacturing was carried out by 'one or two processing and manufacturing complexes supplying the six member states with cigarettes' [22]. However, to function efficiently, this would require the abolition of customs duties among member states. The report concluded that the best scenario, from BAT's point of view, would be 'a single market without internal customs duty tariffs but with a high external duty tariff on cigarettes ... That would provide the best condition for rationalisation of processing, manufacture and supply with protection against imports' [22]. A later report noted that, although this would also give PM 'further opportunities' to expand through the supply of the Honduran and Nicaraguan markets from Costa Rica, Guatemala or El Salvador, BAT would be able to respond on the basis of its 'marketing knowledge, local experience and... ability to offer superior product quality and financial muscle'[23].

Documents describe how BAT envisioned its goals were to be achieved by having cigarettes that were imported from outside the CACM placed on a list of products to be given special treatment and which would thus be allowed to have a tariff higher than 20%. However, the lower tariff of 5% was preferred by BAT for raw materials and goods used in the manufacture of cigarettes, given the need for the company to import these cheaply. BAT saw it as crucial that 'internal trade barriers be removed before the reduction in external tariffs'. If this were not the case, its national operating companies would face outside competition before being able to rationalise their manufacturing operations along the lines described above. Operating companies were to lobby both the CACM's permanent secretariat, the Secretariat for Central American Economic Integration (SIECA), and 'local' (i.e. national) ministers, 'arguing that the reduced external tariffs would threaten a long established regional industry that is not only a substantial provider of employment, but also an increasingly important generator of foreign exchange'[22].

#### Access to government: Nicaragua

As a major company and employer in the countries of the CACM, the extent of access that BAT had at this time to governments in the region is evinced by a visit Sir Patrick Sheehy (BAT Chairman) and his wife paid to Nicaragua in April 1991. After the visit, they expressed their pleasure at having met Mrs Violeta Chamorro (President of Nicaragua), 'and so many members of the government' [24]. By January 1992 the company was already making progress, with a reduction in excise in Nicaragua from 42% of retail sale price (RSP) to 40% and an increase in the selective consumption tax which Nicaragua levied on imported cigarettes from 60% to 75%. It was reported by Keith Dunt (BAT Regional Director for Latin America) that the former 'represents at first calculation US$1.2 million extra contribution in 1992', presumably to company revenue, whilst the latter 'puts us on a par with product imported from Central America and has an advantage on product imported from outside Central America' [25] (BAT's attempts to influence excise taxes are discussed more fully below).

#### Access to government: Costa Rica

Documents suggest a similarly close relationship between BAT and the Costa Rican government. A March 1992 report described meetings with the Ministry of Exports and the Ministry of Economy where employees of BAT's Costa Rican subsidiary, Republic Tobacco, were informed about the negotiating positions of other CACM countries and even given advice on how to lobby them [26]. Named Guatemalan officials had opposed the 5% common external tariff on acetate tow (purified wood pulp used in cigarette filters) and cigarette paper, which BAT supported at this low rate as an importer of these inputs. It was reported that, 'Local [Costa Rican] authorities recommended strong lobbying [by BAT] in Guatemala on this issue', whilst Republic Tobacco would 'continue our lobbying efforts with members of the Costarican (sic) Negotiating Group' [26]. A technical meeting attended by all the CACM Directors of Integration was due to be held at the end of March or beginning of April, which was seen as 'an excellent opportunity to discuss this issue', and Republic Tobacco was preparing a presentation to be given to the Ministry of the Economy prior to this meeting [26]. The document also described the issue of imported tobacco additives (casings and flavours) for which an El Salvadoran representative was insisting on a 10% tax. Again, 'local [Costa Rican] authorities suggested strong lobbying' of the Director of Economic Integration in El Salvador to remedy this [26]. In September 1992 it was reported that 'the CACM negotiators approved the request of the Local Tobacco Industry to reduce from 10% to 5% the taxes on cigarette paper, filter tipping, plugwrap, casings and flavours'[27].

#### Coordinating lobbying efforts

As part of its strategy to influence tariff rates, BAT set up an 'information exchange' across all of its Latin American companies, so that data on the 'rapidly evolving and indeed changing CACM tariff regime' could be circulated on a weekly basis [28]. National operating companies were instructed to 'report active intelligence through contacts in governments and trade organisations, however tentative this might be', while general managers were asked to 'ensure that a proactive stance in lobbying was developed and a positive programme developed for each company'[29]. Guidance given to Republic Tobacco suggested that, in preparing its case for 'bureaucratic and government officials involved in negotiations for CACM treaty terms', each company or industry NMA (National Manufacturers' Association) 'should itemise what is at risk on the local scene and state that cheap [cigarette] imports would not be dissuaded by the 20% import tariff... and this therefore would mean a loss of foreign exchange as the economies of scale outside give significant lower costs of production'[30].

More detailed advice followed in which the need to 'lobby strongly' on tariff levels and to give the matter 'priority in any discussions with Government' was emphasised [31]. Among the key arguments to be used were that the 'cigarette industry makes a significant contribution to Government revenues', with the instruction that this should be 'quantif[ied] in any discussion', and that the 'collection of Government revenue through the domestic cigarette industry is secure and timely - this would not be the case if imported cigarettes both duty paid and duty not paid begin to penetrate your domestic market'[31]. It was suggested that the European Community be used as a comparison, since at the time it had 'a common external tariff of 90% for imported cigarettes' [31]. It was further instructed that governments should be presented

with clear worked examples demonstrating that with such low international prices and with a tariff structure of only 20%, the future of domestic cigarette manufacturing rapidly becomes unviable. Domestic duty paid cigarettes would soon be unable to compete with imported cigarettes either on price or quality. The impact of this scenario on the Government must be stressed:

e.g. - outflow of foreign exchange with increased imports

- less reliable collection of revenue

- lower revenue as cigarette prices fall

- impact on employment within the industry and to tobacco growers and local suppliers since there will be less demand for leaf and other services for domestic purposes. [[31], emphasis in original]

Regional coordination of lobbying efforts was increased still further in October 1992 at a public affairs coordination meeting. It was decided that all public affairs managers in the region should send to Republic Tobacco details of all taxes being paid on raw materials. From this information, a list would be prepared of 'all raw materials with taxes above 5% to coordinate lobbying efforts in the region' [32]. BAT headquarters at Millbank in London was to prepare 'a position paper on the route to follow on tax harmonisation' [32]. The circulation of information was deemed insufficient, sometimes involving the simple circulation of summaries of newspaper articles. This was to be remedied by ensuring that public affairs managers obtained 'first hand information directly from the sources in order to have updated information and closer links for effective lobbying' [32]. In order to 'ensure a regional approach for effective lobbying, it was agreed that a coordinator for CACM countries would be appointed by Millbank' [32].

#### Targeting tariff rates

By November 1992, BAT had fixed its target tariff rate for cigarette imports at 60%, three times the common external tariff that CACM countries initially intended to implement in January 1993. An early achievement was reported in BAT's internal public affairs review: 'In Costa Rica the Ministers of Agriculture, Foreign Trade and the Ministry of the Presidency agreed to back the industry positioning to increase the external tariffs from 20 per cent to 60 per cent and to include cigarettes and tobacco in the CACM free trade agreement' [33]. BAT's regional business review reported the same month that 'BATCo has a unique lobbying synergy and has been actively making representations to the respective Governments and the Secretariat (SIECA) to exclude cigarettes [from the 20% common external tariff] and fix a 60% import tariff for the region'[34].

While the common external tariff of 20% was not implemented by the initial CACM target date of 1 January 1993, the tariff rates for cigarettes imported from outside the region had been reduced by that month to between 20% to 30%, from up to 40% previously [35] (see Table 2), prompting BAT to intensify its lobbying further. Also in January 1993, the company's Honduran subsidiary reported that the National Association of Industrialists had been persuaded to back its campaign to raise the tariff, and would 'request from government that cigarettes be excluded from the products lists subject to the common external tariff' [36]. Success came in February when a meeting in Guatemala of the CACM Directors of Economic Integration included cigarettes and tobacco on a list of products that could have tariffs above the 20% level [37]. Dunt wrote to the General Managers of BAT's Central American companies to congratulate them: 'This is clear evidence of what we can achieve when we co-ordinate well together and act on a common platform throughout the region. It is now obviously key to us to plan the next stage with detail and again on a common platform'[38]. Each company was required to prepare a proposal on the tariff levels for a meeting of the Directors of Economic Integration to take place in March 1993. This meeting agreed cigarette tariffs ranging from 30% to 75% and thus substantially above those of 20% to 30% operating in January 1993, although generally below BAT's now higher target rate of 75% (see Table 2) [39]. The 30% to 75% tariff levels agreed in March 1993 were the maximum permitted by the CACM countries' commitments under the General Agreement on Tariffs and Trade (GATT) (which varied by country), so any implementation of tariffs above these levels would need to be renegotiated within the GATT process [35,39,40]. These new rates also still had to be ratified by the CACM Duty Council, and this appears to have been postponed for a substantial period while countries grappled with the implications of a further tariff rise for their commitments under GATT.

**Table 2:** Percentage tariff rates on cigarettes imported into CACM countries from outside the region [Sources: [35,39,47]]

Country	Tariffs prior to January 1993	Tariffs in January 1993	Tariffs agreed in March 1993	Common External Tariff 1996
Costa Rica	40	27	55-40*	55

Guatemala	40	30	30	55

Honduras	30	20	45	55

Nicaragua	20	Unknown	75	55

El Salvador	20	20	35	55

*Under its GATT commitments, Costa Rica was initially expected to reduce its tariffs on cigarettes to 40% by January 1995, but could levy a tariff of 55% until then.

Nonetheless, this represented a significant achievement for BAT. Indeed, the audacity of BAT's campaign on the external tariff issue is attested to by the fact that it continued to press CACM governments to attempt further renegotiation of these GATT 'ceilings'. Documents suggest member countries agreed to do so by negotiating for a 'uniform regional consolidated ceiling' during the Uruguay Round of the GATT [39,41]. At the end of December 1993 it was reported that:

Lobbying to increase the common external tariff on imported cigarettes into Central America has achieved an agreement in principle from all the Region's finance ministers to increase the tariff to each country's agreed GATT level and to then approach GATT and seek an increase to a level of 75%. Each country needs to ratify the increase to the GATT level, and this is awaited. Ex-GATT expertise is now involved in the preparation of the companies' arguments[42].

Documents describe how BAT was heavily involved in this process, with consistent access to relevant government ministers. The extent of this access is summarised in a 1993 'Public Affairs Review' in which managers of BAT's various Central American subsidiaries described 'the overall status of... government relations' as follows:

Costa Rica: We have ready access and close contact with government officials at all levels.

El Salvador: Good government relations with Ministers (Economy and Treasury), Congressmen (party leaders), Ministry technicians.

Guatemala: Very good relations. General Manager and Public Affairs Manager know most Officials and Ministers personally. This includes the President and Vice President of the Republic.

Honduras: The government has an excellent opinion of TAHSA [the BAT subsidiary]. The President of the Republic visited us some months ago and expressed his sympathy for the way we conduct business. When PA Manager has asked for benefits from the Government, the answer has usually been positive. I would say the relations are excellent.

Nicaragua: TANIC [the BAT subsidiary] has good relations with all key Government departments on an informal basis... [43]

In February 1994 Edgar Cordero (Corporate Affairs Director, TANIC), who was coordinating BAT's lobbying in the CACM, 'visited the Nicaraguan negotiator in GATT from the Ministry of Economy and also the Director of C.A. [Central American] Integration to discuss progress on GATT, Free Trade Agreements and CET [common external tariff]' [44]. However, the difficulty of renegotiating tariffs within GATT was then made clear when Nicaragua 'officially received three major objections to its intention of consolidating its ceiling (75% in the case of cigarettes)', from the United States, Canada and the European Community [45].

Available documents are incomplete in their coverage of this issue and it is thus unclear how these objections were resolved in GATT. However, documents describe BAT arguing for a lower level of common external tariff of 55% by September 1995: 'Efforts have continued to raise the Central American Common Market External Tariff for cigarettes to 55%. Costa Rica has implemented this level, and other CACM Agriculture Ministers have agreed to put this forward to their governments'[46]. A further document indicates that this 55% level had been agreed by 1996: 'The Central American Ministers of Economy ... approved the request of BAT companies to reduce the tariff on [leaf] tobaccos imported from outside the CACM while maintaining the 55% external tariff for cigarettes'[47]. In the same year, the CACM governments agreed to reduce the tariff on crucial imported inputs to cigarette manufacturing: 'Following discussions, CACM governments have agreed to reduce import duties for raw materials from 5% to 1% including cigarette paper, tipping [the paper surrounding a cigarette filter], tow, etc'[48].

### 'Continuing our aggressive lobbying campaign at all government levels': Influencing excise taxes

As well as influencing tariff rates, BAT was concerned with how excise taxes (taxes on goods produced for sale, or sold, within a country) impacted on the regional tobacco trade. A report circulated within BAT in October 1991 noted that, in 'a perfect Common Market excise and other similar taxes on goods would be harmonised' so as to allow goods to move freely between the member countries without any fiscal distortions to trade [22]. Although there were no plans to harmonise excise or other domestic taxes at that time, BAT's analysis of country excise taxes concluded that 'the rates all lie between about 30% and 42.5% of RSP [Retail Sale Price], which means that harmonisation, if it was ever contemplated, would not be very difficult'[22]. Subsequently, Guatemala, El Salvador and Honduras formed 'the Northern Triangle', whose declared aim was the creation of a true common market [11], and in March 1993 they proposed to standardise excise rates among them. In a letter to managers of BAT's subsidiaries in these countries, BAT employee Alastair Young appraised the momentum towards standardisation as 'fragmented' [49]. He recommended that, whilst BAT should not oppose any excise reduction, it should limit its lobbying on the issue and 'delay the process of standardisation until we are in a position to manage and control the changes' [49]. This could be done by arguing that, 'Standardising an inappropriate system of Excise is no solution - we can assist the authorities in developing a system - but this needs time'[49].

By May, however, Paul Bingham (BAT Director of Marketing) concluded that 'BAT is marginally late into resolving this issue but ahead of Philip Morris and not too late to influence the outcome of harmonisation discussions'[50]. Noting that '[g]overnments, SICA [Central American Integration System], SIECA [Secretariat for Central American Economic Integration] are open to BAT lobbying', Bingham laid out a schedule for coordinated lobbying by the various operating companies across the region, drafted from BAT headquarters and precise to the day, but which should be adapted to the sensitivities of each national context:

May 24: Bingham to draft out the position paper and send to all GM's [general managers] and Finance Directors (to be sent on 24/5).

May 28: Rosales to adapt Bingham position paper to the sensitivities of the region and issue to all CACM countries.

June 5: Each Op Co [operating company] to prepare a national position paper based upon the overall paper but including appropriate national aspects.

June 12: Implementation of national lobbying to ensure acceptance of the BAT approach to harmonisation [50].

The position paper would propose the following:

- structural harmonisation only based upon a % of the retail price.

- a minimum absolute excise to prevent price wars and cheap imports.

- maintenance of existing [tax] burden in each country [50].

Documents suggest that the levying of excise on the retail sale price (RSP), rather than on the ex-factory price (i.e. the price paid at the factory and not including any transport costs), was important to BAT because the latter would give a relative advantage to imported cigarettes, since it would not include importers' margins and distribution costs [49,51]. Bingham concluded his letter by referring to a previous visit to the region: 'Although I had only one political meeting (Vice Minister of Finance: El Salvador: E Montenegro), the Bingham b******t on excise seemed to go down well'[50].

In July 1993 Graham Burgess (Head of Planning, BATCo) reported that, 'A major lobbying effort to change and unify the excise structure in six Central American countries is underway... The Technical Advisory Group to the CA [Central American] Finance Ministers on this issue appears to be sympathetic to BAT's recommended excise structure for the region'[52]. BAT's ability to lobby across the CACM countries in a coordinated manner was underlined in its El Salvadoran company plan for 1994-1996:

Excise taxes and import duties will be under review within the forming Central American Common Market... We will grasp this opportunity by continuing our aggressive lobbying campaign at all government levels (technicians, negotiators, legislators and Ministers) to ensure a satisfactory outcome for the industry (high common import duty, low materials duties and low excise duties). This will be coordinated with other C.A. companies to ensure we capitalize our unique lobbying power through of (sic) having an operating company in each C.A. country [53].

The company plan for Guatemala made clear the role of central direction in this coordinated lobbying effort: 'Maintain a constant direct monitoring of government and congress activities on new taxes, tariffs and excise structures and values procuring information from the source, *following strategies from Corporate Affairs Department in Millbank*'[[54], emphasis added].

By November 1994 documents describe BAT's lobbying campaign showing results:

SIECA, the technical advisory group to the Central American Common Market is preparing a recommendation to the Council of Finance Ministers of the CACM. The proposal will follow the recommendation from BAT CA i.e. a move to harmonise the excise structure in the CACM based on RSP (and not ex-factory price)[55].

In September 1995 it was reported that a harmonised excise structure had been agreed by the CACM vice-ministers of finance, although ratification of this remained pending [46].

### Reorganising BAT's operations

BAT's strategy in response to the CACM integration process had two crucial components, each of which was necessary in order to allow the other to work effectively. As described above, the first was the maintenance of a high common external tariff for cigarettes, a low common external tariff for leaf and other inputs into the production process, and low or non-existent internal tariffs between member countries. The second component was the rationalisation of its operations to take advantage of regional economies of scale. The first component was crucial to the working of the second, since high external tariffs on cigarettes were necessary to protect its regional dominance from outside competition, whilst the lowering or removal of internal tariffs resulting from the integration process created an opportunity to take advantage of economies of scale, by reducing the number of production centres and distributing from them to the other countries of the region. Importantly, successful implementation of this second component was necessary to enable BAT to compete effectively with PM, which would also be protected by higher external tariffs and advantaged by the larger economies of scale afforded by the reduction of internal tariffs. The latter was particularly important for PM, which could now potentially challenge BAT in those CACM countries where it had no production facilities, through cheap imports from other CACM countries. BAT's assessment of PM's likely strategy was as follows:

Guatemala will continue to develop as the centre of PM activities in the CACM, specifically as the central production source. Surplus funds generated in Guatemala will be used to finance PM expansion into Honduras and Nicaragua, and a more aggressive competitive posture generally. Guatemala is therefore an important strategic market that has significant implications for further PM expansion in the region [56].

PM's operation in Guatemala, the only country in Central America where it had the largest market share, was 'the designated PM factory for Central America', making it 'well structured for the development of CACM'[57]. It was, therefore, deemed 'imperative that BAT improves its share position in order to fully occupy PM in the domestic market' [56], whilst putting in place its own plans for regional reorganisation. Although BAT had the largest market share in most Central American countries, this share had tended to decrease as a result of competition from PM, which dominated the US international brands segment through its focus on Marlboro. BAT anticipated that PM would utilise 'regional resources across markets', including 'integrating in C.A. to initiate attacks on BAT's dominance in Honduras and Nicaragua'[58]. Without a concerted effort by BAT, the company's prognosis was '[r]educing share, volumes and profits for BATCo' [59].

BAT's plan for reorganising its Central American operations was named the 'Central America Initiative', which was 'designed to glean productivity and efficiencies out of treating Central America more as a region for BAT than as six free standing independent companies'[28]. Continuing to manage its business in Central America 'with six stand-alone companies' was seen as 'a recipe for decline' and would expose the company 'to increasing threat from PM, who is already integrating (sic)' [60].

BAT's Central American companies would thus move towards 'a centralised management structure' with 'a regional top functional team... headed by a regional GM [General Manager]. Management structures in the operating companies will be changed to reflect the regional management concept, resulting in significant reductions in international staff and total staffing over the plan period' [61].

Leaf operations were the first to be rationalised, with the implementation of a 'single management structure' and 'single leaf strategy' in place of the pre-existing national operations, in order to 'consolidate operations, reduce costs and gain sustained competitive advantage from the synergies obtained'[62]. As one document explained: 'Central America has an urgent need to put in place a single sourcing strategy to replace the current territory-by-territory approach which is effectively yielding product quality and cost disadvantage viz-a-viz our competitors'[63]. The rationalisation of leaf operations was particularly important due to a significant reduction in demand for exported leaf, following measures adopted by the US to protect its tobacco farmers.

The crux of the reorganisation, however, was the rationalisation of manufacturing operations. The initial time schedule for this was set out in BAT's company plan for 1995-1997, which envisaged the closure of factories in Guatemala, Honduras, Nicaragua and Panama between October 1994 and December 1996, with all production moving to El Salvador and Costa Rica [64]. Under the plan, operating company general managers would be converted into 'country managers', who would manage sales and distribution in their country where there was no production facility [65].

The closure of the Guatemalan factory was the first step, and a detailed document on this provides insight into how the rationalisation plans would be implemented. It was noted that 'the implementation of this rationalisation project would depend mainly on its marketing and political implications rather than on technical issues'[51]. However, the political fallout from the closure was thought to be manageable, since the Guatemalan subsidiary had already succeeded in reducing its leaf operations:

The political, economic and social impact of this closure has been evaluated and is considered to be manageable. TANSA [BAT's Guatemalan subsidiary] has already implemented, in 1993 and 1994, two restructuring exercises to downsize its leaf operation in response to the decline in leaf export demand. 24 TANSA employees were made redundant and the number of farmers was reduced from 1,189 to 160. There was no negative reaction[51].

The factory closure would leave the Guatemalan operation to focus 'on the core activity of cigarette marketing and distribution' and would involve a number of redundancies: '36 employees would be made redundant immediately, 8 employees would be offered transfers to other departments, and 5 employees would be retained temporarily (assuming they accept) to supervise machinery withdrawal etc. and then subsequently to be made redundant'[51]. It was envisioned that this would take place in 'one step', since a 'prolonged two step closure increases the risk of the union being reactivated, or other employee actions to prevent implementation' [51]. A redundancy package was put in place to help to achieve the 'key closure implementation objective [of] prevent[ing] the re-emergence of unionization', and contingency plans to keep sales operations running and ensure the safety of senior management were put in place in case of labour unrest [51]. Although BAT calculated that 'the decision will be accepted as commercially justified and a consequence of the CACM development' [51], the substantial redundancies resulting from regional rationalisation were directly at odds with BAT lobbying arguments that a high common external tariff was needed to protect employment, described above. Indeed, total employment in BAT's Central American operations fell from 2,208 in 1992 to 1,722 in 1996, even before the closure of other factories [58].

As rationalisation progressed, BAT decided to centralise production even more than originally envisaged. Instead of the region being supplied from factories in El Salvador and Costa Rica, all production would be consolidated in one expanded factory in Honduras, as part of a 'global manufacturing strategy' to increase volume and quality as efficiently as possible [66-68]. This global strategy to rationalise production was itself allied to the worldwide growth of free trade areas: 'As with competitors, British American Tobacco's key area of manufacturing cost saving is likely to be achieved by taking advantage of free trade areas to take radical steps to consolidate production into large factories' [69].

## Discussion

The documents reviewed in this article demonstrate the degree of access BAT had to decision-makers in Central American countries during negotiation of the CACM and, in particular, the extent to which it was able to influence decisions on tariff rates and excise taxes. Furthermore, the company was able to exploit its multinational character to act in a coordinated way across the member countries of the CACM. Through these means, BAT was able to have the tariff rate for cigarettes imported into CACM countries set higher than would otherwise have been the case, and to play a key role in harmonising excise taxes across member countries. It did this by utilising high-level contacts with politicians and key officials, pooling information across national units in the region, and stressing the importance of the company to employment, tax revenues and foreign exchange within each country. Each national operating company was given strong central direction by the regional management at BAT headquarters to ensure that lobbying was conducted in a coordinated way around these core arguments.

Whilst the degree of access to governments and officials in the countries and institutions of the CACM is striking, it is consistent with previous studies of TTC policy influence in a number of other settings. Indeed, the degree of such access and the extent to which policy makers appeared to take for granted the importance of the industry to their economies may have made some of the tactics used elsewhere unnecessary. For example, we found no evidence in the documents we reviewed of donations to political parties or legislators' campaigns, although it is important to note that the documents of BAT's Central American subsidiaries, other than correspondence with its UK companies, were not available. Furthermore, information on these matters from official sources is not as widely available in the countries concerned as it is in the US and we could not therefore verify from other sources whether such donations had been made. What is of particular note in this case is the degree to which BAT was able to act transnationally in a coordinated manner across all the CACM countries simultaneously and the way in which central direction of the various operating companies was used to gather intelligence and ensure consistency and optimum timing in the messages communicated to governments.

While it was making its arguments to governments, documents show that the company began to reorganise its own operations to take advantage of the economies of scale afforded by the reduction of internal tariffs within the CACM, protected by high external cigarette tariffs, in a manner that benefited the company on a regional basis rather than any particular country. In direct contradiction to the arguments presented to CACM governments that affording the tobacco industry protection via high tariffs would safeguard employment, the company's regional reorganisation involved the loss of hundreds of jobs.

This loss of employment was a logical component of the company's strategy of regional rationalisation which it was planning even whilst it was lobbying governments on the basis of protecting jobs. In fact, this rationalisation is in keeping with the logic of regional trade integration itself, which depends for its envisaged efficiency enhancing effects in part upon the economies of scale facilitated by the abolition or reduction of internal tariffs, and BAT's behaviour in this respect was mirrored by PM, with which it had to compete. CACM member governments themselves would presumably have anticipated such processes in at least some industrial sectors, which perhaps explains why BAT thought the process would be 'accepted as commercially justified'. Nevertheless, it took concerted action to manage the 'political implications' of the rationalisation process and put in place substantial contingency plans in case of labour unrest.

With the process of regional trade integration within the CACM being one of many set in train during the 1990s, BAT strategy within the CACM has significance beyond Central America. Indeed, BAT documents identify such processes as a crucial factor leading the company to reorganise globally in the 1990s, so that it might act in a more coordinated manner across countries to increase worldwide sales and thus consumption of its cigarettes [2].

However, this article suggests the company's activities in no way indicate an ideological commitment to any general process of liberalisation. Rather, findings indicate that BAT's responses to processes of regional integration have been purely instrumental; that is, it has acted in whatever manner would most protect and advance the company's interests in a specific context. In the CACM, this meant pressing for higher external tariffs for cigarettes, low tariffs for imported inputs necessary for cigarette production, and an excise tax system that best fitted its interests. This is consistent with its behaviour in other settings. In Uzbekistan, for example, the company sought protective tariffs, an excise system that benefited it rather than its competitors, and other anti-competitive measures [4,70]. It is different, however, to its behaviour in the Andean Pact, where similar processes of regional trade integration led the company to recognise that it had failed to effectively coordinate imports into countries where it did not have production facilities, subsequently leading it to reorganise these activities in that region and across Latin America [2]. In East Asia in the 1980s and 1990s, US-based TTCs (including BAT's American subsidiary Brown & Williamson) pressed hard for liberalisation in a number of countries in order to gain access to markets from which they were excluded [71]. In the CACM, emphasis was also placed on coordinated action across the region. However, as BAT had a factory in each member country, it sought to gain regional economies of scale from the rationalisation of production within the region. In contrast to Asia in the 1980s and 1990s, the company pushed for high external cigarette tariffs because it already dominated the region and wished to protect its market share. Whilst these findings confirm previous analyses that TTCs regularly attempt to influence trade policy, they suggest that responses to liberalisation processes across companies and contexts are not uniform. These different responses indicate the need for further research to better understand the strategies of particular tobacco companies in seeking to influence trade policy in other regional trade negotiations such as those of the Common Market of the South (Mercosur), the North American Free Trade Agreement (NAFTA) and the Association of South East Asian Nations (ASEAN) Free Trade Area (AFTA).

Although a significant number of documents arising from correspondence between BAT's UK companies and its Central American subsidiaries were available through the Legacy Tobacco Documents Library, the study was limited by gaps in the available documents, particularly those of the company's Central American subsidiaries. Similarly, a relatively small number of documents relevant to the study were available after 1995. This meant that it was not possible to ascertain how the issue of tariff ceilings was resolved within the GATT or how the harmonised excise structure agreed by the CACM vice-ministers of finance in September 1995 progressed.

## Conclusions

Effective responses to protect public health require a fuller understanding of the complex dynamics between tobacco and trade policy. Existing analyses of this relationship have largely focused on the role of trade liberalisation in increasing competition and therefore the consumption of cigarettes. This article indicates that trade liberalisation at the regional level can have quite different impacts on market structure and strategy. The impact of regional trade integration on tariff and tax rates, manufacturing operations and corporate organisation within the tobacco industry has not been widely studied, yet is integral to the transnational behaviour of tobacco companies and their capacity to continue to thrive in a rapidly changing world economy. The need to better understand this dimension of trade and tobacco is especially important given the shift in trade negotiations following the stalling of the Doha Round from multilateral (i.e. the World Trade Organisation) to regional and bilateral venues.

Regional integration organisations and their member states should be wary of the capacity of TTCs to influence negotiations in ways that serve corporate, rather than member state, interests. These findings also suggest the need to ensure that public health goals are adequately coordinated at the regional level. This could be achieved, for example, by addressing regional trade integration at regional and subregional workshops on the implementation of the decisions of the Conference of the Parties of the Framework Convention on Tobacco Control (FCTC) [72]. Through such mechanisms, measures for countering the region-specific strategies of TTCs, such as those focused on taxation and tariff rates, could then be developed.

## Competing interests

The authors declare that they have no competing interests.

## Authors' contributions

CH conceived of the study, searched, analyzed and interpreted documents, wrote the first draft of the manuscript and revised subsequent drafts. KL helped to conceive of the study, contributed to the analysis and interpretation of findings and helped to edit the manuscript. Both authors read and approved the final manuscript.
